# Editorial: Optimizing player health, recovery, and performance in basketball

**DOI:** 10.3389/fpsyg.2022.1101052

**Published:** 2022-12-02

**Authors:** Davide Ferioli, Daniele Conte, Aaron T. Scanlan

**Affiliations:** ^1^Research Center for High Performance Sport, UCAM Universidad Católica de Murcia, Murcia, Spain; ^2^Department of Movement, Human and Health Sciences, University of Rome “Foro Italico”, Rome, Italy; ^3^Institute of Sport Science and Innovations, Lithuanian Sports University, Kaunas, Lithuania; ^4^School of Health, Medical and Applied Sciences, Central Queensland University, Rockhampton, QLD, Australia

**Keywords:** assessment, fitness, game-related statistics, injuries, load monitoring, performance analysis, training strategies, technical performance

Basketball is one of the most popular team sports globally, with participation rates ranging from 2 to 5% among adults (aged >18 years), 7–14% among adolescents (aged 13–17 years), and 5–25% among children (aged 5–12 years) in African, American, and Western Pacific regions (Hulteen et al., 2017). This participation rate has grown recently in many countries—for example, 27.1 million people from the United States over 6 years of age participated in basketball in 2021 compared to 22.3 million in 2016 (Statista, 2022). Furthermore, basketball is played across many competitive levels ranging from recreational settings to international tournaments such as the Olympics. In line with this broad appeal and increased participation, the number of journal publications focused on basketball has grown in the past 20 years (Figure 1), placing it second in publication outputs among Olympic team sports (Millet et al., 2021). Consequently, this Research Topic, *Optimizing player health, recovery, and performance in basketball*, was conceptualized as an outlet for this increased scientific interest to further strengthen the available evidence base for basketball end-users.

**Figure 1:**
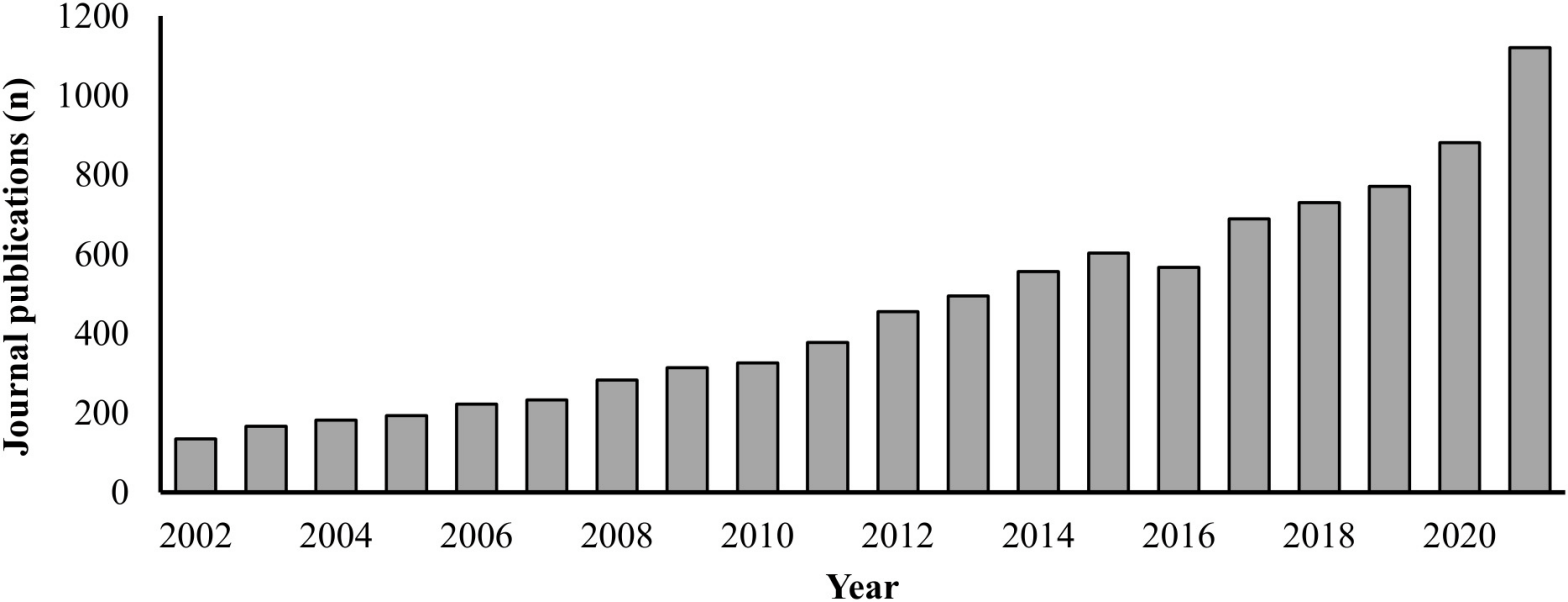
Growth in the number of basketball Scopus-indexed journal publications between 2002 and 2021. Search conducted using Scopus on 27 October 2022 for “basketball” within “Article title, Abstract, Keywords” field, with “Journal” selected as source type and “Article in Press” excluded.

The development of relevant research questions that meet the needs of end-users and provide real-world impact is essential to evidence-based practice (Fullagar et al., 2019a). In this way, the different focal areas of this Research Topic (i.e., player health, recovery, and performance) align with preferences for research evidence among end-users working in competitive sport (Fullagar et al., 2019b; Schwarz et al., 2021). For instance, most surveyed practitioners employed within a sports organization (at the collegiate, professional, or Olympic level) in the United States (*n* = 67, with 16% working in basketball) indicated they used research evidence for health-related functions [injury prevention (91%), nutrition (85%), and rehabilitation (81%)], recovery (94%), and performance-related functions [fitness (79%) and load monitoring (73%)], with research contributing most to developing individualized preparation/recovery strategies and optimizing individual performance (Fullagar et al., 2019a). Furthermore, most of the basketball literature has been identified to focus on topics related to physiology (Millet et al., 2021), injury (Scanlan and Dalbo, 2019; Millet et al., 2021), testing/assessment (Millet et al., 2021), load monitoring (Scanlan and Dalbo, 2019), and game statistics (Scanlan and Dalbo, 2019), which likely encompasses various health-, recovery-, and performance-related research questions. Consequently, the studies published in this Research Topic provide novel evidence in areas that are relevant to basketball end-users by extending upon popularized areas and expanding areas in need of further attention such as technical/tactical components and skill acquisition (Fullagar et al., 2019a).

Three studies published in this Research Topic focused on external load monitoring among basketball players. Player monitoring is commonly employed by basketball practitioners (Fox et al., 2020), with external load data indicating what players do and being an integral part of the physical training process to impact health, recovery, and performance outcomes among players (Jeffries et al., 2022). Firstly, Russell et al. provide the most comprehensive analysis of external loads imposed upon a National Basketball Association (NBA) team to date, reporting demands during different tasks according to player role, experience, and position across a season. Secondly, Stone et al. provide insight into the utility of different external load variables measured using microsensors according to position among male, National Collegiate Athletic Association Division I players. Thirdly, Pernigoni et al. used video-based time-motion and microsensor technologies to quantify the demands experienced during jumps, sprints, and high-intensity specific movements, as well as with and without ball possession according to position among semi-professional, male basketball players.

Four further descriptive studies focused on quantifying anthropometric, fitness, behavioral, or technical/tactical attributes among basketball players, generating evidence that may inform practical strategies in health- and performance-related areas including player assessment, selection, and nutrition. Firstly, Sato et al. described the associations between facial width-to-height ratio measurements and performance during games (i.e., efficiency rating) among professional, male basketball players. Secondly, Popowczak et al. highlighted the importance of anthropometrical attributes when elucidating associations between physical and cognitive variables during reactive agility and change-of-direction speed tests in professional, female basketball players. Thirdly, Rösch et al. concluded that the Basketball Learning and Performance Assessment Instrument possessed adequate reliability in assessing various performance and technical variables but lacked diagnostic validity in identifying selected (vs. non-selected) youth (under-15 years), male players within a national program. Fourthly, Sánchez-Díaz et al. demonstrated male players had superior physical fitness and led more active lifestyles than female players, with all players possessing inadequate nutritional habits and knowledge among youth (under-14 years) players from a national program.

An additional three studies examined training strategies and statistical indicators in relation to technical performance, team performance, and injury rate in basketball players. Firstly, Caparrós et al. demonstrated that irrespective of the strength training program undertaken, strength variables alongside muscle injuries were associated with team performance outcomes in professional, male basketball players. Secondly, Milley and Ouellette showed that using an external focus of attention imagery technique benefited free-throw shooting performance compared to an internal focus of attention strategy in collegiate basketball players. Thirdly, Yi et al. concluded that various game-related statistics including two-point field goal percentage, offensive rebounds, assists, and turnovers were key for team success in professional, female players.

The collection of studies presented in this Research Topic cover various areas encompassing player samples spanning across sexes, ages, and competitive levels. This openness to research among basketball teams is encouraging and should be nurtured through the continued development of studies that are symbiotic for stakeholders in terms of implementation and outcomes. Accordingly, dedicated work is advocated to ensure the most impactful questions are developed in future studies (Buchheit, 2016) and identify how the generated outcomes can be most effectively disseminated for implementation (Buchheit, 2017)—which is yet to be explored specifically among basketball practitioners. In this way, embedding research students (*via* partnership with institutions) or research staff (e.g., sport scientists) within basketball teams may assist in ensuring not only the most relevant evidence is generated, but also effectively communicated and implemented (Fullagar et al., 2019a). Of note, no studies in this Research Topic examined 3 × 3 basketball, which should be given greater attention moving forward given its rapid rise in global popularity and recognition as an official Olympic sport at the recent Tokyo 2020 games. Furthermore, innovations to technology and strategies that reduce injury/illness risk, enhance return-to-play progression, improve the recovery process during specific seasonal phases, and promote desired performance levels should continue to be developed and tested *via* empirical research to ensure we are continually optimizing player health, recovery, and performance in basketball.

## Author contributions

AS and DF wrote the original draft of this editorial, while DC reviewed and edited it. All authors have approved the final version of this editorial.

## Conflict of interest

The authors declare that the research was conducted in the absence of any commercial or financial relationships that could be construed as a potential conflict of interest.

## Publisher's note

All claims expressed in this article are solely those of the authors and do not necessarily represent those of their affiliated organizations, or those of the publisher, the editors and the reviewers. Any product that may be evaluated in this article, or claim that may be made by its manufacturer, is not guaranteed or endorsed by the publisher.
